# “The message hurts, but it would be worse if nothing was said” – Communicating severe illness and the imminence of death to patients with end stage heart failure and their families – A longitudinal interview study

**DOI:** 10.1371/journal.pone.0328993

**Published:** 2025-07-23

**Authors:** Annika Pohl, Maria Liljeroos, Tiny Jaarsma

**Affiliations:** 1 Physician in Family Medicine and Palliative Medicine, LAH Norrköping, Vrinnevi Hospital, Norrköping, Sweden; 2 Department of Health, Medicine and Caring Sciences, Linköping University, Linköping, Sweden; 3 Centre for Clinical Research Sörmland, Uppsala University, Eskilstuna, Sweden; Kwame Nkrumah University of Science and Technology, GHANA

## Abstract

**Aims:**

Discussions about severe illness and the coming death do not often take place with patients with heart failure and their family. We therefore aimed to investigate how patients with end-stage heart failure and their family who discussed terminal illness and the imminence of death with a physician, experienced such communication, how they handled life emotionally and practically after said discussions, and if/how this changed over time.

**Methods:**

A longitudinal interview study. Ten patients with end-stage heart failure and their closest kin were visited by a physician at home and discussed terminal illness during one visit and the imminence of death during another visit. They were interviewed three times about how they experienced the communication and how they handled life in this situation and in relation to the discussions. The interviews were analysed using qualitative thematic analysis by Braun and Clarke.

**Findings:**

Two main themes and five subthemes were found. The first theme was ‘an honest and clear message hurts, but it would be worse if nothing was said’, and the subthemes included information on the experiences of communication, the desired level of communication by patients and family members and factors facilitating communication. The second theme was ‘A clear message helps in handling life’ with the subthemes of coping psychologically and practically. The findings indicate that for some patients and family members it was hard to have discussions about end-stage heart failure and the imminence of death. However, they found the discussions important and were happy that the information was not withheld from them. The discussions helped in handling life and most patients and family seemed to have found a way to accept and handle the situation. Practical planning often did not start until they heard from the physician that death could come soon.

**Conclusion:**

This study confirms that patients and family members want and appreciate discussions about severe illness and the imminence of death and find them important. This can encourage physicians to change behaviour and engage in honest discussions, and to educate and train colleagues to do the same.

## Introduction

Information about end-stage heart failure, poor prognosis, and imminent death is often not given to patients and their families. Discussion about advanced care planning (pertaining to decisions regarding do not resuscitate orders and referral to palliative care), are seldom held with patients with terminal heart failure and their family [1,2]. This even though such communication is strongly recommended in professional guidelines (e.g., by the European Society of Cardiology) and that it is known that most patients wish to receive candid but compassionate communication about these issues [3–8].

Previous research has shown there are several reasons why communication about end-of-life does not take place [1,5,6,9,10]. Patients and families do not always know that heart failure is a disease leading to death [1,6], making them less prepared to face a poor prognosis [9]. Talking about end-of-life can be a difficult task due to the uncertain prognosis and the unpredictable heart failure trajectory [1,5,10]. In patients with heart failure, death can occur during the current period of deterioration or the next period of deterioration—or suddenly, in between active periods of deterioration [3,11,12]. Organisational issues like discontinuity of care between the physician doing the follow-up and those who see the patients when they are seriously ill in the emergency room [13], and limited time for communication, also negatively affect end-of-life-communication [10,14]. Physicians often are unsure of how to discuss these matters and are afraid that they could provoke anxiety and take hope away from their patients [1,5,10,12].

If patients are unaware of their prognosis or their life expectancy, they might go to the emergency room and the hospital late in their disease progression, and undergo advanced and sometimes exhausting investigations and treatments that do not help, and they may not receive optimal symptom relief [15,16]. Without knowledge about their prognosis or disease trajectory, patients may not be able to plan for end-of -life; for example make plans about where they want to spend their last period in life and what contacts they want or need to make before life is over [4,17]. Awareness of poor prognosis and that death can come soon are necessary for advance care planning and for referral to specialist palliative care [12,15,18,19]. Currently, only few patients with terminal heart failure receive palliative care even though they have a symptom burden comparable to patients with cancer [12,20,21].

Patients generally prefer to be informed by healthcare providers in ways that are clear, compassionate, and proactive [22]. In a review, Low and colleagues called for an improvement in coordination of care and communication between patients with heart failure, their families, and health care professionals. They also concluded that there may be differences in views between the disciplines of cardiology and palliative care regarding differences in particularly the maintenance of life-prolonging treatment as goals of care change [23].

Avoiding honest communications can make patients and family less likely to understand the illness of the patient, and this might result in increased psychological suffering with more anxiety, depression, confusion or having unrealistic expectations from health care [4,5,17,24,25]. Awareness of prognosis is associated with greater satisfaction with care [24].

Given that most patients do not receive communication about severe illness and imminent death, the aim of this study was to investigate how patients with end-stage heart failure and their family, who discussed terminal illness and the imminence of death during two home-visits with their physician:

1. Experienced the communication with the physician
2. Coped with life between and after the discussions
3. Whether/how experience of the communication and coping strategies changed over time.

## Methods

### Study design and setting

The study had a qualitative design with data collected during interviews with patients with end-stage heart failure and their family members. The setting is in Swedish health care. There are no specific protocols in Sweden for delivering information about end stage heart failure or that provide guidelines for communication. Some centres have physicians that are specialised in palliative medicine, and in a few centres, there are palliative care units for heart failure and pulmonary diseases.

This study took place in a centre with a physician specialised in palliative medicine and trained to talk to patients about end-stage heart failure and the imminence of death. Data was collected during, before, in between, and after communication with this physician. Data were analysed using qualitative content analysis [26]. Data were collected from December 2018 to July 2020.

### Study population and sampling procedure

Patients were identified in the internal medicine department in a hospital or in primary care in southeast Sweden. Medical records were reviewed by the first author to identify potential patients. We also invited family members of the patients who were included in the study.

#### Patient selection.

**Inclusion criteria:** Diagnosed heart failure, NYHA IIIb-IV (verified by a cardiologist), age ≥ 30 years, and able to communicate in Swedish.

**Exclusion criteria:** Comorbidity with risk of death in the current year—for example, progressive cancer, later-stage COPD (chronic obstructive pulmonary disease), renal failure with eGFR < 15, dementia, or other noticeable cognitive insufficiency (Mini Mental Test was performed when in doubt of a patient’s cognitive function, and patients scoring under 24 points were not included), or serious psychiatric illnesses like schizophrenia or major depression.

Patients were informed about the study by a physician who asked whether a research nurse could contact them to give them additional information about the study. They were invited to be interviewed about how they experience discussions about their disease and its consequences, and how these discussions affect them.

Potentially eligible patients were contacted by a research nurse to be given more detailed information of the study, and to obtain written consent. The patient was asked for consent to approach a family member and then the same procedure was done with a chosen family member.

Fifteen patients were invited, 13 agreed to participate and two patients declined. Three patients started the study, but due to rapid deterioration they could only participate in the first interview. Their interviews are not included in the analysis, so in total we included data of 10 patients in the analysis. Eight family members were included, since two patients did not have close kin.

### Data collection

Medical and demographic data were collected from the medical records. See Table 1.

**Table 1:** Characteristics of patients in the study and interviews performed.

Patient	Age	Gender	Civil state	Caring support	Duration of heart failure	Family member interviewed	Pat I1*	Pat I2*	Pat I3*	Fam I1*	Fam I2*	Fam I3*
1	90	man	married	homecare	4.5	Son	x	x	x	x	x	x
2	85	man	partner	no	7	Partner	x	x	x	x	x	x
3	83	man	married	no	8	Wife	x	x	x	x	x	x
4	87	woman	widow	homecare	11	No	x	x	x			
5	91	woman	married	homecare	5	Son	x	x	x	x	x	x
6	84	man	married	no	2.5	Wife and daughter	x	x	x	x	x	x
7**	82	woman	widow	homecare	3	No	x	x				
8***	85	man	married	no	3	Wife	x		x	x		x
9	76	woman	married	homecare	9	Husband	x	x	x	x	x	x
10	75	man	widower	nursing home	31	Daughter	x	x	x	x	x	x

* Pat I1 = first interview with patient, Pat I2 second interview with patient etc. Fam I1 first interview with family member etc.

** Patient 7 felt well so there was no reason to prepare for the death when the analysis started and therefore there are only two interviews with her.

*** Patient 8 was so ill at the first physician visit so there are only two interviews with him and his wife.

#### Procedure.

**Fig 1:**
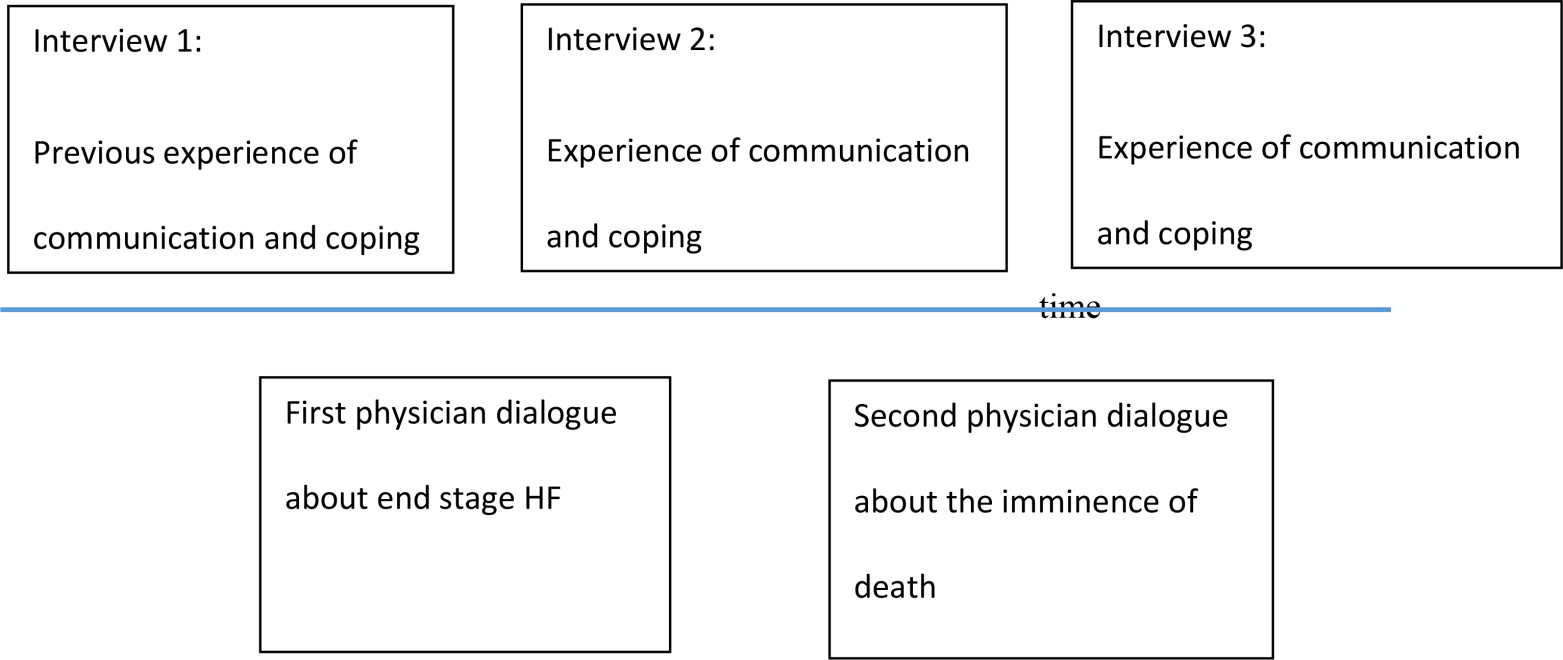
Data collection and physician dialogues with patient and family.

Documented visits by the physician (AP) (see Fig 1) were undertaken with the chosen family member present (if available). This author was not involved in performing the interviews.

#### Physician visits.

During the first visit, the terminal phase of heart failure and its accompanying poor prognosis were discussed. A do not resuscitate order was put into the patient´s medical chart and the patient and their family were informed. If this had already been done, the decision was confirmed. Ordinary clinical duties like history and physical examination were performed. The patient was invited to participate in specialised palliative homecare, and all patients accepted that offer.

The second visit by the physician occurred when death was imminent, which in heart failure patients has to be performed early, as there is much uncertainty regarding timing and disease evolution [15]. The visit included a discussion about the imminence of death, and wishes, thoughts, and preparations for death.

The first author (AP) performed all visits, to make sure the discussions with participants addressed the subjects in an open and clear manner. If needed, extra physician visits were held.

#### Interviews.

Semi-structured interviews were performed before and after the two doctor visits. A nurse who was not involved in the care of the patient performed the interviews. The interviews were conducted with the patient and afterwards with the family member and were held in the patients’ home or at another location convenient for the patient and their family member. The nurse who conducted the interviews has a master’s degree in nursing and has 15 years of experience in palliative care.

The interviewer asked open questions and follow up questions and let patients and family talk until they stopped, even if they strayed outside the subject matter at hand. The length of the interviews varied between 30–60 minutes. The same key questions were asked in the three interviews to allow for the ability to follow up what happens over time; to note whether the experience and/or coping strategies changed. The interviewer wrote field notes after the interviews regarding what feelings she recognised in the room during the interviews.

#### Data collecting tool – Interview guide.

The interview guide (Table 2) was inspired by previous studies [6,7], and addressed experiences with getting information, their needs for more information, their feelings and coping with the received information.

**Table 2:** Interview guide.

What do you know about:
Your heart disease?
The expected course of the disease?
How healthcare will look going forward?
**Who has given you this information?**
**How did you experience receiving such information?**
**Do you want to know more?**
**What do you want to know?**
Who would you like to be informed by? How? When?
**How do you feel about what you know about your disease state?**
Do you feel worried/anxious/depressed/uncertain about the future?
What do you do about this? What helps?
**What do you think your family knows about your state of disease?**
What do you want them to know?
Can you talk about these things together?
**Has this information/communication influenced your life?**
Practically, emotionally and/or in relationships?

### Data analysis

Interviews were transcribed verbatim and analysed using qualitative thematic analysis [26] by the first author (AP) and second author (ML). ML is a cardiac nurse, a post doc researcher and experienced in qualitative methodology. Braun and Clarke`s six phase guide was used for the thematic analysis.

1) Interviews were read (some were also listened to) repeatedly by AP and MLs independently to gain an in-depth understanding of the data.
2) Each transcript was broken down to manageable sections and categorised into codes, to capture the essence of the data.
3) Codes that resembled similar ideas were grouped to subthemes.
4) AP and ML were involved in searching subthemes and themes. This process helped guarantee that the subthemes and themes reflected well the data content.
5) The names of the themes were discussed by all three authors so that they communicated the essence of the findings.
6) Producing the report. Special attention was paid to deviant cases—cases that contradicted patterns of emergent ideas and concepts.

### Rigour

To ensure trustworthiness we provided transparency in methods. The study employs a longitudinal interview design, which is described in detail to allows readers to understand how the study was conducted. We also provide a thorough explanation of the procedures used, including the interview process and interview guide. Data were analysed following the phases of Braun and Clarke. To ensure credibility we worked in a team with relevant backgrounds in palliative medicine, cardiology and nursing. We included direct quotes from patients and family members adds authenticity to the findings, providing a rich, qualitative insight into their experiences.

### Ethics

The investigation confirms with the principles outlined in the Declaration of Helsinki [27] and was approved by the Regional Ethics Review Board in Linköping, Sweden (Dnr 2017/580-31). Confidentiality of the collected data was ensured, and the audio files and written data were securely stored in password-protected computers.

## Findings

Two themes and five subthemes describe patients’ and family members’ experiences discussing the terminal phase of heart failure and the imminence of death with a physician and how patients and family members handled life after these discussions (Table 3).

**Table 3:** Themes and subthemes.

Themes	Subthemes
An honest and clear message hurts, but it would be worse if nothing was said	Experience of communication/discussions
	Patients’ and family members’ level of desired communication
	Factors facilitating communication
A clear message helps in handling life	Coping psychologically
	Coping practically

### An honest and clear message hurts but it would be worse if nothing was said

#### Experiences of communication/discussions.

Most patients reported receiving ambiguous and varied information from different physicians and a lack of continuity for a long time before they received clear communication about end-stage heart failure and their poor prognosis. Patient 9 reported: *“I have been to the emergency room many times and the doctors in primary care are changed every three months. The doctors gave me different messages*—*both that there was nothing wrong and that test results were not good.”* Family member 9 said: *“The communication about her sickness was inadequate before our last visit to the hospital. The doctors have had different opinions and earlier they did not understand what disease she suffered from. It feels afterwards that they should have taken her problems more seriously earlier”*.

Some of the patients had discussed end-stage heart failure before the study and already understood their situation. Some of them could point out the exact time and place where they learned they had end-stage heart failure; were they got the information they could rely on. They also reported they were grateful to have this information. Patient 3 said: *“*She (the young doctor in the emergency room) *said to me, my wife and my oldest son, that it sometimes can go fast and sometimes takes more time, but there was not much that could be done about it. She explained everything well and was not in a hurry. She said, she did not want to lie but to instead tell the truth. She hoped things would go well, but said we had to accept what comes.”* Patient 8 reported: *“I have wanted to get information and got it without concealment. He (the physician in cardiology) gave me good information, allowed me to ask my questions and he took his time. So, I am well prepared and feel secure in having it all planned.”* Participants described these meetings with their physician as part of a good relationship, where a compassionate and honest meeting took place. One of the patients, patient 10, who already knew his prognosis, commented: *“He* (the cardiologist on the ward) *told me every day that the prognosis was bad. It is not exactly what you want to hear all the time. I knew that. It was too much information*”.

Most patients described that they were satisfied that they came to know about their condition from their physician during these discussions. Some patients pointed out that at first it was difficult to receive the message that they had end-stage heart failure and/or that they probably only had a short time left to live. At the same time, they said they did not want the information to be withheld from them. Several patients and family members pointed out how important it was to receive the message, although it did hurt. They said it in different words with the same meaning*: “The message hurts but it would be worse if nothing was said”* (Patients 1, 3–5, 8–10).

Patients and family appreciated honest communication about end-stage heart failure and their poor prognosis. Patient 10 said*: “She was very clear about the bad prognosis; she said it was best to put off the internal cardiac defibrillator to make it less painful in case of heart arrhythmia ending my life. She was clear that death can come fast or that there can be some time left. I appreciated it that she said it in a simple and straightforward way and that she was coming back.”*

Patient 9, after a discussion about how death can come soon, commented: *“In a way it feels good to know life can be over any time, to know that this is the time I have, and in a way it is tough. It feels unreal*—*I try to take it in. I can, however, not say I am unprepared; I have talked to the children, and my funeral is planned.”*

#### Patients’ and family members’ level of desired communication.

None of the patients said they did not want to receive this kind of communication, even if it sometimes felt difficult. Two family members were reluctant for the physician to talk about the terminal phase of the illness or the coming death with the patients. One of them, a son, family member 1, changed his mind after the discussion had taken place and then said: *“the more you know, the better.”* One spouse, family member 2, felt disappointed after the discussion in the fact that death could come soon. She did not want her partner to know: *“I saw that he was sad after the conversation. I then thought she could have talked to me in the kitchen. I would not tell him.”*

Two couples did not speak to each other about severe illness and the imminence of death. Patient 2 said: *“I will be alone at the end. No one can cope with this.”* Patient 6 reported: *“I am worried about what will happen to my wife if something happens. We have not talked about it*—*only thought about it.”*

#### Factors facilitating communication.

Factors influencing successful communication were reported from patients and family members. It is good when the doctor informs the patient in an open, clear, and honest manner in a language that is easy to understand, that the doctor is present emotionally, is understanding, takes time, follows up, and does what can be done. A wife, family member 3, said*: “The doctor asked if I was worried about him. Some doctors talk and you do not understand a thing, and some talk so you can understand the meaning of what they say.”* Patients and family noted that it is also possible to communicate openly and clearly when there is an uncertainty of the timing of the disease progression. Patient 1 said: *“It is better to be honest about the unsure parts than fake certainty and be proven wrong.”*

### A clear message helps in handling life

#### Coping psychologically.

After the discussions about severe illness and/or the imminence of death, sometimes after a short reaction and processing time, most patients and family seem to have found a way to accept and handle the situation. Recurrent comments were: *“I try to take one day at a time”*(Patients 1,2,3,4,6,10); *”I cannot do anything about it so I do not put my energy into it”* (Patients 3,4,6,10); *“When that day comes, the only thing you can do is go along with it”*(Patients 1,2,3,4,5,6); and *“I am rather satisfied with my life”*(Patients 4,5,8,9). Some patients and family gave a more detailed description of their feelings: patient 9 said: *“I am surprised that I think so little about it and that I am so calm. In the big picture, I am satisfied with my life.”* Her husband, family member 9, commented: *“I am now used to the thought that my wife will die before me. I have been prepared and realise the seriousness, we talk about death, and I have a grieving process that has been going on for some time. I think I should be tormented by grief over what is happening, but I am not, I accept it. I cannot do anything about it.”*

Many patients described that they did not think about how sick they were, or about death, when they felt good. Both patients 1 and 10 said: *“I do not think of the hard bits all the time, but I do not close my eyes to it.”*

Patients and family pointed out that the fact that death can come soon, must be talked about, and described their reactions. A daughter, family member 10, said: *“It was tough. I understand that this is serious. It was a good dialogue; it would have been worse if nothing were said. Every day we have together is a gift and this is very obvious right now.”* Patient 4 described: *“When she said you can die at any moment it gets obvious. That feels a little hard. But they have to tell me, so I am prepared.”*

Some family members were especially grateful to know that they have a short time left in life. Sometimes patients feel that death will come soon, but family members do not have that knowledge. Patient 5 commented: *“I have felt myself becoming worse, I have nearly a total lack of energy.”* Family members wanted to be prepared and know that the patient’s time was near so as to make the best use of the time they had left.

Some patients and family members commented that communication about how death could come soon was a good reminder about their insecure and critical situation, because after some time it is easy to live as if there was no threat at all, and it encouraged them to make practical decisions.

#### Coping practically.

When death can come soon it is important to confirm that to the patient and family, since practical planning for many does not start until this is done. Patient 4 said: *“I did know that it was bad, but not so bad. When you hear the words, it gets more real. I think about my cats and planning for the funeral. It is hard but if you have to, you have to.”* Nearly all, both patients and family, took practical steps after that discussion to plan for the funeral, to plan their epitaph or memorial, and to make arrangements for pets. Family members asked the patient questions which they have found difficult to ask, before it became obvious that arrangements were now necessary.

## Discussion

Our study found that open and clear communication about severe illness, poor prognosis, and that death can come soon was important for patients and their closest family members, confirming previous studies [4–6]. The patients and nearly all family members in this study wanted to have these discussions. Some were reluctant at first, but afterward, felt grateful.

Studies performed in patients with heart failure show that they want this kind of information but seldom receive it [1,4,6,12,14,15,28]. It is important to address how to improve the frequency and quality of these discussions.

Education, training, tutoring and role modelling are needed to overcome the uncertainty of physicians about how and when to discuss serious illness and end-of life issues [10,12,29–32]. They have to overcome the fear of making patients and families upset and taking the hope away and learn to do these conversations in a compassionate way [1,5,10,12]. Ensuring support from health care that they are not being left alone is important [24]. Physicians need to practise the skill of explaining clinical uncertainty to patients who will likely die soon [5].

Better professional communication skills can help patients and family to understand their situation, facilitate advance care planning and make patients and family more ready to face the realities of end-of-life [31]. Choosing words wisely when heart failure advances can help patient and family emotionally accept that it is now time for end-of-life care [33]. Emotional support by professionals and using compassionate silence can enable a clinician to affirm mutual respect and understanding [34,35]. Collaboration between cardiology and palliative care and the inclusion of each other’s expertise in professional education is needed to learn from each other [14,21,28,36,37]. In different specialities of medicine there are different cultures. Generally stated, cardiologists may focus mainly to extend life as long as possible and might find it harder to acknowledge the need for a ‘change in gear’ in their treatment for patients who are at the end of life. It can be one reason that the communication about severe illness and the coming death might be underdeveloped in patients with heart failure. [12].

The longitudinal approach in this study is unique, following both patients and family over time with three interviews surrounding the time they talked with the physician about terminal illness and the imminence of death. The patients in our study had a variety in disease trajectories and had received different information before discussions occurred about end-stage heart failure prognosis. For most patients and their family members, discussions about end-stage heart failure and bad prognoses were new, which was reflected in evolving reactions over the course of the study, when the same questions were asked three times. Others who already had these discussions with their physicians prior to the study sometimes already processed this information.

Another important strength in this study is that we interviewed both patients and family members. Family members can often feel neglected and anxious when their ill family member is near the end of life. Family members can have a distinct need of information [24]. Creating opportunity for open one-to-one communication between family caregiver and health care professionals is vital to enable family to prepare for what lies ahead at the end of life and prevent feelings of abandonment after the death of the patient [38]. The feeling of being supported is related to being involved in decision making and being aware of the poor prognosis [38]. Furthermore, as the severity of heart failure progresses over time, caregiving demands on families are often expected to change and intensify over time [39]. The value of having both patients and their closest family members receive the same message and able to ask questions can help reduce insecurity [19,40].

Although the longitudinal design with integrated data from both patients and family is a strength of the study, some limitations should be considered.

One limitation to this study is that only Swedish-speaking patients were included from a certain region in Sweden. We do not know whether these reactions/experiences would be the same in other cultures or language groups. Another limitation is that we could not interview all patients several times due to the sudden deterioration or do member checks, since patients were already dead when transcribing and analysing data.

## Conclusion

This study contributes to existing research by interviewing patients and family members who received discussions about severe illness and the imminence of death and confirms that patients and family members want and appreciate these discussions and find them important. Currently, such discussions do not routinely take place, although it is known that most patients want this honest communication. This can encourage physicians to change behaviour and engage in honest discussions, and to educate and train colleagues to do the same. Future research might focus on the optimal timing for these discussions.
